# Avoidance, biomass and survival response of soil dwelling (endogeic) earthworms to OECD artificial soil: potential implications for earthworm ecotoxicology

**DOI:** 10.1007/s10646-017-1788-1

**Published:** 2017-03-09

**Authors:** C. Brami, A. R. Glover, K. R. Butt, C. N. Lowe

**Affiliations:** School of Forensic and Applied Sciences, University of Central Lancashire, Preston, PR1 2HE UK

**Keywords:** Artificial soil, Avoidance test, Ecotoxicology, Soil dwelling earthworms

## Abstract

Soil dwelling earthworms are now adopted more widely in ecotoxicology, so it is vital to establish if standardised test parameters remain applicable. The main aim of this study was to determine the influence of OECD artificial soil on selected soil-dwelling, endogeic earthworm species. In an initial experiment, biomass change in mature *Allolobophora chlorotica* was recorded in Standard OECD Artificial Soil (AS) and also in Kettering Loam (KL). In a second experiment, avoidance behaviour was recorded in a linear gradient with varying proportions of AS and KL (100% AS, 75% AS + 25% KL, 50% KS + 50% KL, 25% AS + 75% KL, 100% KL) with either *A. chlorotica* or *Octolasion cyaneum.* Results showed a significant decrease in *A. chlorotica* biomass in AS relative to KL, and in the linear gradient, both earthworm species preferentially occupied sections containing higher proportions of KL over AS. Soil texture and specifically % composition and particle size of sand are proposed as key factors that influenced observed results. This research suggests that more suitable substrates are required for ecotoxicology tests with soil dwelling earthworms.

## Introduction

The potential for earthworms as bio-indicators of environmental quality is widely recognised (reviewed by Fründ et al. 2011). Litter dwelling (compost) earthworms have been widely adopted for use in both acute and chronic ecotoxicological studies, with *Eisenia fetida* proposed in a number of standardised tests (OECD Acute Toxicity Test (OECD 1984), USEPA OCSPP 850.3100 Earthworm Sub-chronic toxicity test (USEPA 2012), ISO 11268-1:2012 acute toxicity test (ISO 2015) and ISO 17512-1:2008 avoidance test (ISO 2012). This is due to a short life cycle, high fecundity, relative ease of cultivation and commercial availability. However, the use of such species in ecotoxicology has been openly questioned (Lukkari et al. 2005; Lowe and Butt 2007) as they do not inhabit mineral soil, have a limited distribution associated with naturally occurring organic matter and are therefore considered to have limited ecological relevance. The use of soil dwelling species is increasingly advocated (Svendsen et al. 2005; Suthar et al. 2008; Butt and Lowe 2011) particularly as perceived issues associated with maintenance and culture have been overcome (Lowe and Butt 2005).

Artificial soils are often preferred to natural soils in standardised toxicity tests as they allow conformity in, and comparability of results, are available throughout the year and do not contain organisms or pollutants that can influence the test. In standardised earthworm-related tests (e.g. ISO 17512-1:2008, ISO 11268-1:2012), OECD artificial soil (AS) is recommended. However, the use of OECD AS may not always be appropriate. Hofman et al. (2009) refer to several specific issues including: (1) validity of test result extrapolation to field conditions, as the properties of OECD AS are substantially different to natural soils; (2) variation in toxicity results between laboratories employing OECD AS, as the specific properties of each component are not specified, even though the component composition of AS is strictly defined. A number of researchers have sought to address the second issue (Bouwman 2007), however such studies have focussed on development of substrates for epigeic earthworm species.

Several studies (e.g. Shoults-Wilson et al. 2011; Loureiro et al. 2005) have found that avoidance of contaminants by earthworms can be equivalent to or more sensitive than traditional endpoints, such as biomass gain/loss and mortality. Only one standardized avoidance test (ISO 17512-1, 2008) has been developed and recommends the use of *E. fetida* and *E. andrei*. This standard details the methods for a two-section and also a six-section avoidance test, with the latter difficult to set up and rarely used. Lowe et al. (2016) developed an avoidance test that allows for the establishment of a linear pollution gradient within rectangular mesocosms (troughs) that are simpler to establish than the six-section chamber test and also allow for a larger range of concentrations than the two-section chamber design.

As soil dwelling earthworms are adopted more widely in ecotoxicology, it is important to establish if standardised test parameters remain applicable. Two-section avoidance tests have been used to study the influence of soil properties (Natal-da-Luz et al. 2008), by manipulating OECD AS, but these have focused on epigeic rather than soil dwelling earthworms. The main aim of this study was to establish the influence of OECD artificial soil on soil-dwelling (endogeic) species (*Allolobophora chlorotica* and *Octolasion cyaneum*) in terms of survival, change in biomass and avoidance behaviour (utilising a linear gradient rather than a two-section methodology).

## Materials and methods

An initial experiment investigated the influence of two soil types on survival and change in biomass of mature *A. chlorotica.* A standardised OECD artificial soil (AS) was established; with a composition of: Sphagnum Peat 10%, Quartz sand 69.5%, Kaolinite Clay 20%, Calcium Carbonate 0.5%. For comparison, Kettering loam (KL) a natural soil that is widely used in earthworm-related studies (see Lowe and Butt 2005) was obtained from Boughton Loam Ltd (KL composition: Clay 24%, Silt 18%, Sand 58%, Organic content 6.72%; pH 6.8).

Six replicates of each soil treatment were set up in opaque plastic containers (0.07 m × 0.05 m × 0.07 m), with lids pierced with a mounted needle to allow ventilation. Dried (at 105 °C) and sieved horse manure (2 g per 100 g of soil) was incorporated into each soil as a feed source and the substrate rewetted to a moisture content of approximately 25% (Lowe and Butt 2005). Each container was filled to a depth of 0.04 m with the relevant soil treatment. Mature *A. chlorotica* had their mass determined (mean biomass (±s.e.) = 0.249 ± 0.013 and 0.225 ± 0.013 g in KL and AS respectively) with two individuals placed into each container. Thereafter, containers were maintained in 24 h darkness at 15 °C in a temperature-controlled incubator (considered by Lowe and Butt (2005) to be optimal conditions for the culture of temperate soil-dwelling earthworms). Treatments were sampled after 14 and 28 days, with mass re-determined.

In a second experiment, the influence of soil type (AS, KL) on avoidance behaviour of *A. chlorotica* and *O. cyaneum* (mean biomass 0.31 and 0.44 g respectively) was investigated utilising the methodology of Lowe et al. (2016). A gradient was established with five soil treatments which were combinations of AS and KL viz: 100% AS, 75% AS + 25% KL, 50% AS + 50% KL, 25% AS + 75% KL and 100% KL. Dried and sieved horse manure (2 g per 100 g of soil) was incorporated into each soil treatment as a feed source and the substrate rewetted to a moisture content of approximately 25%. Equal volumes (0.12 m × 0.135 m × 0.085 m) of each soil treatment were established and initially separated by plastic spacers (cut with a laser to the dimensions of the mesocosm).

A single earthworm was placed on the surface of each soil treatment (*n* = 5 earthworms per mesocosm) and after introduction, the spacers were removed and the containers covered with plastic (cling) film, pierced (with a mounted needle) to allow ventilation and kept in 24 h darkness at 15 °C. Five replicates were established for each earthworm species. After 14 days, containers were carefully removed from the incubators, spacers re-inserted and earthworm positions within soil gradients determined by destructive sampling.

Statistical analyses were performed with Minitab software (Version 17). In the initial experiment, differences in *A. chlorotica* biomass were assessed using a student’s t-test. In the avoidance experiment a Kruskal Wallis test followed by a Mann-Whitney post-hoc test executed with a Bonferroni correction was utilised to compare earthworm retrieval rates.

## Results and discussion

**Fig. 1:**
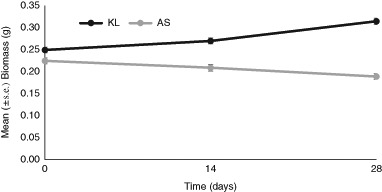
Change in mean biomass (+/−standard error) of A. chlorotica in Kettering Loam (KL), and Artificial Soil (AS) over 28 days

*A. chlorotica* survival after 28 days in the initial experiment was 100 and 91.7% in KL and AS treatments respectively. At day 0, there was no significant difference (*p* = 0.206) in the mean biomass of *A. chlorotica* in the AS and KL treatments. After 14 and 28 days, individuals in AS had a mean biomass of 0.209 and 0.189 g respectively, significantly lower (*p* < 0.05) than individuals present in KL (0.270 and 0.315 g respectively) (Fig. 1).

In the avoidance experiment, 100 and 95% survival of *A. chlorotica* and *O. cyaneum*, respectively was recorded after 14 days, therefore both avoidance tests results are considered valid as less than 10% of individuals were missing or dead (ISO 17512-1: 2008).

**Fig. 2:**
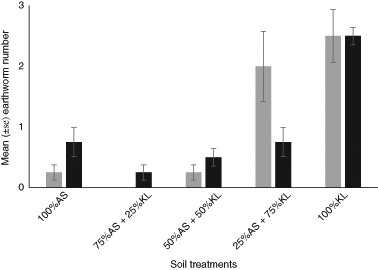
Mean (±standard error) number of A. chlorotica (*grey bar*) and O. cyaneum (*black bar*) recorded in each section of the gradient, filled with different proportions of Kettering Loam (KL) and Artificial Soil (AS), after 14 days

For *A. chlorotica*, significantly more (*p* < 0.05) earthworms were retrieved from both 100% KL and 25% AS + 75% KL than from the three other soil treatments (Fig. 2). For *O. cyaneum*, the mean number recovered from 100% KL was more than three times greater than in the four other soil treatments (Fig. 2).

Our results demonstrated that the OECD artificial soil resulted in a loss of *A. chlorotica* biomass over a 28 day period and that both *A. chlorotica* and *O. cyaenum* selectively avoided AS when compared with KL in a linear, proportional soil gradient. It is likely that these findings can be directly attributed to differences in the physical and chemical properties of the soil types. While further research is required to establish the exact nature of these observations, the authors consider it appropriate to highlight the potential influence of soil texture.

Soil texture is known to affect soil properties such as cation exchange capacity, nutrient status and soil moisture so can have an important influence on earthworm populations (Edwards and Bohlen 1996). During development of OECD AS, the high sand content was intended to produce a “worst case scenario” with respect to the bioavailability of contaminants in acute toxicity tests (Hofman et al. 2009). However, earthworms, and in particular soil-dwelling species, are rarely found in sandy soils. Several authors (e.g. Al-Yousef and Shoreit 1992; Hendrix et al. 1992; Baker et al. 1998) have shown a strong correlation between earthworm abundance and soil texture with earthworm populations positively correlated with soil clay content. Furthermore, Baker et al. (1992) and El-Duweini and Ghabbour (1965) found that the number and mass of *Aporrectodea trapezoides* and *A. caliginosa* (endogeic species) were negatively correlated with the sand and gravel content of soil. Sand texture may also influence earthworm behaviour. As soft bodied organisms, earthworms are particularly sensitive to coarse particles within the substrate and this may elicit a behavioural response. As an example, Kretzschmar (1991) advocated coating the inside of experimental vessels with sealing varnish and sharp fine sand to prevent *Aporrectodea longa* burrowing in the space between the vessel wall and the soil. Artificial Soil has a sand composition of 69.5% and comprised an industrial quartz sand with a requirement that particle size does not exceed 2 mm (0.6–2 mm considered coarse). While Kettering loam also has a relatively high sand composition (58%), only 1% of sand has a particle size between 1–2 mm, while 53% has a particle size under 0.5 mm (Grundy 2015). As soil dwelling endogeic species burrow through the soil profile and are also geophagous, differences in soil texture may influence both burrowing and feeding behaviour. In the context of current standardised toxicity tests, it is important to note that soil texture would have minimal influence on epigeic species that in general do not inhabit mineral soil.

Hofman et al. (2009) posed the question “Is the OECD artificial soil really a standardised reference material omitting the influences of varying soil properties?” While the current research did not address this question directly, it does add to concerns regarding continued use of OECD AS in earthworm ecotoxicology. However, further work is needed to clarify these results and establish suitable acceptable test substrates for soil and litter dwelling earthworms.

Finally, our work has demonstrated the effectiveness and potential sensitivity of the linear gradient methodology in assessing earthworm avoidance behaviour. This may have specific applications in measuring the potential effects of emerging soil contaminants, such as nanomaterials, that are currently present at low concentrations within the soil matrix.
